# Effects of 12 weeks of beta-hydroxy-beta-methylbutyrate free acid gel supplementation on muscle mass, strength, and power in resistance trained individuals

**DOI:** 10.1186/1550-2783-9-S1-P5

**Published:** 2012-11-19

**Authors:** KA Dunsmore, Ryan P Lowery, NM Duncan, GS Davis, JA Rathmacher, SM Baier, EM Sikorski, TJ Morrison, MA Naimo, J Walters, Stephanie MC Wilson, Jacob M Wilson

**Affiliations:** 1 Department of Health Sciences and Human Performance, The University of Tampa, Tampa FL, USA; 2 Metabolic Technologies, Inc., Iowa State University Research Park, Ames, IA, USA; 3 Department of Animal Science, Iowa State University, Ames, IA, USA; 4 Department of Biology, The University of Tampa, Tampa FL, USA; 5 Department of Nutrition, IMG Performance Institute, IMG Academies, Bradenton, FL, USA

## Background

Previous research in trained individuals supplemented with beta-hydroxy-beta-methylbutyrate (HMB) has been constrained to short (<10 weeks), non-periodized studies, lacking dietary control, that were subject to poor outcome measures (e.g. skin caliper measurements). These conditions make it difficult to determine HMB’s effects in athletes. The primary purpose of this study was to investigate the effects of 12 weeks of HMB free acid (HMB-FA) supplementation in trained individuals on direct skeletal muscle hypertrophy (ultrasound muscle thickness), strength, and power during periodized resistance training.

## Methods

Twenty resistance trained males (21.3 ± 1.9 years) were randomly assigned to consume 3 g per day of HMB-FA (combined with food-grade orange flavors and sweeteners) or a placebo (food-grade orange flavors and sweeteners) in a double blind manner. All subjects participated in 12 week periodized resistance training consisting of full body workouts centered around the squat, bench press, and deadlift, and auxiliary exercises of pullups, military presses, bent over rows, barbell curls and extensions. Volume and intensity undulated such that Monday, Wednesday, and Friday subjects performed hypertrophy (3 sets of 8-12 RM loads and 60 seconds rest), power (3-5 sets of 1-5 repetitions, 40-60 % 1-RM loads, 2-3 minutes rest), and strength (3-5 sets of 1-5 RM loads, with 3-5 minutes rest) respectively for weeks 1-8. This was followed by 2 weeks of an overreaching, pure hypertrophy training on M-TH, and strength on Friday. The final two weeks, subjects tapered (50-80 % volume reduction) while focusing on strength and power. All subjects were placed on a diet consisting of 25 % protein, 50 % carbohydrates, and 25 % fat by a registered dietician who specialized in sport (RD, LDN, CISSN). Subjects total strength (squat + bench press + deadlift), power, and muscle mass of the quadriceps were measured at 0, 4, 8, and 12 weeks. Data were analyzed with a 2 X 4 repeated measures ANOVA with LSD post hoc tests utilized to determine where differences occurred.

## Results

There were no differences in total calories, protein, carbohydrate, or fat consumed between groups. There were time, and group x time effects (p<0.05) for total strength, which increased by a greater percentage in the HMB (430.4 ± 22.5 to 507.5 ± 21.7 kg; + 18.3 %) than the placebo group (422.2± 24.9 to 447.5 ± 22.5 kg; + 6.6 %). There were time, and group x time effects (p<0.05) for Wingate peak power, which increased to a greater extent in the HMB (876.6 ± 46.0 to 1035.5 ± 55.7 watts; + 21.9 %) than the placebo group (882.9± 50.8 to 986.3 ± 22.5 kg; + 16.2 %) p<0.05). Finally there were time, and group x time effects (p<0.05) for muscle thickness, which increased to a greater extent in the HMB (50.7 ± 1.6 to 57.8 ± 1.7 cm; + 14.5 %) than the placebo group (49.6± 1.7 to 52.0 ± 1.9 cm; + 4.7 %) (p<0.05).

## Conclusions

In conclusion, these results suggest that an HMB-FA supplement can enhance adaptations in strength, power, and hypertrophy following a 12-week, periodized resistance training program.

